# The association between accelerometer-measured patterns of sedentary time and health risk in children and youth: results from the Canadian Health Measures Survey

**DOI:** 10.1186/1471-2458-13-200

**Published:** 2013-03-07

**Authors:** Rachel C Colley, Didier Garriguet, Ian Janssen, Suzy L Wong, Travis J Saunders, Valerie Carson, Mark S Tremblay

**Affiliations:** 1 Health Analysis Division, Statistics Canada, Ottawa, ON, Canada; 2 Children’s Hospital of Eastern Ontario Research Institute, Ottawa, ON, Canada; 3 School of Kinesiology and Health Studies, Queen’s University, Kingston, ON, Canada; 4 Department of Community Health and Epidemiology, Queen’s University, Kingston, ON, Canada

**Keywords:** Behaviour, Breaks, Bouts, Physical activity, Pediatric

## Abstract

**Background:**

Self-reported screen time is associated with elevated health risk in children and youth; however, research examining the relationship between accelerometer-measured sedentary time and health risk has reported mixed findings. The purpose of this study was to examine the association between accelerometer-measured patterns of sedentary time and health risk in children and youth.

**Methods:**

The results are based on 1,608 children and youth aged 6 to 19 years from the Canadian Health Measures Survey (2007–2009). Sedentary time was measured using the Actical accelerometer. Breaks in sedentary time and prolonged bouts of sedentary time lasting 20 to 120 minutes were derived for all days, weekend days and during the after-school period (i.e., after 3 pm on weekdays). Regression analyses were used to examine the association between patterns of sedentary time and body mass index (BMI), waist circumference, blood pressure and non-HDL cholesterol.

**Results:**

Boys accumulated more sedentary time on weekdays after 3 pm and had a higher number of breaks in sedentary time compared to girls. Overweight/obese boys (aged 6–19 years) accumulated more sedentary time after 3 pm on weekdays (282 vs. 259 min, p < .05) and as prolonged bouts lasting at least 80 minutes (171 vs. 133 min, p < .05) compared to boys who were neither overweight nor obese. Prolonged bouts of sedentary time lasting at least 80 minutes accumulated after 3 pm on weekdays were positively associated with BMI and waist circumference in boys aged 11–14 years (p < .006). Each additional 60 min of sedentary time after 3 pm on weekdays was associated with a 1.4 kg·m^-2^ higher BMI and a 3.4 cm higher waist circumference in 11–14 year old boys. No sedentary pattern variables differed between girls who were not overweight or obese and those who were overweight/obese and none of the sedentary pattern variables were associated with any health markers in girls.

**Conclusions:**

The findings confirm results of other studies that reported accelerometer-measured sedentary time was not associated with health risk in children and youth. Even when the pattern and timing of sedentary time was examined relative to health markers, few associations emerged and were limited to boys aged 11–14 years.

## Background

The association between sedentary behaviour and health risk in children appears to be influenced by how the sedentary behaviour is measured, defined and categorized. Several studies have reported significant associations between self-reported screen time and increased risk of obesity and cardio-metabolic disease risk in children [1-4]. However, screen time provides a limited perspective on total sedentary time because it is only a sub-component of a behaviour that is defined as encompassing “any waking behaviour characterized by an energy expenditure ≤1.5 METs while in a sitting or reclining position” [5]. Further, self-reported data relating to lifestyle habits may be limited by bias and recall difficulties [6,7].

Accelerometers are now commonly used to objectively measure total sedentary time, and have the capacity to also derive the pattern and timing in which it is accumulated. In contrast to self-report, associations between accelerometer-measured sedentary time and health risk in children have been mixed with some studies reporting significant associations [8-10] and others not [1,11-16]. Studies that collected both questionnaire and accelerometer data on sedentary behaviour/time found an association between self- or parent-reported screen time and health risk but no association between accelerometer-measured sedentary time and health risk [1,14,15]. The only studies reporting significant associations between accelerometer-measured sedentary time and health risk did not adjust for moderate-to-vigorous physical activity (MVPA) [8-10] or reported that the significant associations existed in unadjusted models but were attenuated when MVPA was controlled for [13,16]. Some engagement in sedentary behaviour is inevitable in the day (e.g., eating, relaxing, homework, school etc.); however, it is presently unknown how much sedentary time is too much. Inevitably, there is variation between people in the length of time they engage in sedentary behaviour bouts (i.e., prolonged periods of sedentary time) and how often these bouts are interrupted by activity. Research in adults suggests that the pattern of accumulation of sedentary time is important to consider in relation to health risk.

The inconsistent findings between accelerometer-measured sedentary time and health risk among children and youth have led to the examination of more sophisticated sedentary time variables. For example, it has been proposed that the pattern in which sedentary time is accumulated may provide insight beyond what has been observed to-date using the total volume of sedentary time [17]. Others have attempted this approach in adults and children; however, the sedentary pattern variables have been limited to breaks or interruptions in sedentary time [17] and engagement in prolonged bouts of sedentary time lasting up to 30 minutes [1]. Further, studies examining how the pattern of sedentary time relates to health risk in children have not considered the importance of periods of discretionary free time separate from the whole day in children [18-20]. The present study sought to build upon this work by extending the length of the prolonged bouts up to 2 hours and by examining these variables during periods when children and youth typically have free time. In other words, this study sought to identify novel sedentary pattern variables that were more representative of how children and youth typically engage in sedentary behaviour.

The purpose of this study was to examine the association between accelerometer-measured patterns of sedentary time and health risk in children and youth. Specifically, this study examines whether breaks in sedentary time and sedentary time accumulated as prolonged bouts during periods of discretionary free time in children (i.e., after-school and weekends) have stronger associations with health risk in children when compared to average daily sedentary time across the week. We hypothesize that sedentary time accumulated during periods of discretionary free time will better discriminate between children engaging in healthy and unhealthy levels of sedentary behaviour when compared to simply examining overall sedentary time.

## Methods

### Data source

The Canadian Health Measures Survey (CHMS) collected data from a nationally representative sample of the population aged 6 to 79 years living in private households at the time of the survey. Data were collected at 15 sites across Canada from March 2007 through February 2009. Ethics approval was obtained from Health Canada’s Research Ethics Board [21]. For children aged 6–13 years, written informed consent was obtained from a parent or legal guardian, in addition to written informed assent from the child; youth aged ≥14 years provided independent consent. Of the households selected, 69.6% agreed to participate. Of that group, 88.5% of the selected 6–19 year olds completed a questionnaire and 86.9% of this group participated in the mobile examination centre component. Of the children and youth who agreed to wear the accelerometer and returned the device, 87.4% had at least one valid day of data, and 76.3% had at least four valid days. These multiple stages of response can be multiplied together (69.6% × 88.5% × 86.9% × 76.3%) to provide an overall response rate of 40.8%. Adjustments were made at each stage to manage any potential non-response bias. The data were then weighted to be representative of the Canadian population. More extensive details of the CHMS [22] and direct measurement of physical activity in the CHMS [23,24] are available elsewhere.

### Study procedures

Upon completion of the mobile examination centre visit, ambulatory respondents were asked to wear an Actical accelerometer (Phillips – Respironics, Oregon, USA) over their right hip on an elasticized belt during their waking hours for seven consecutive days, except when the device could get wet. The Actical measures and records time-stamped acceleration in all directions, providing an index of physical activity intensity. The Actical has been validated to measure physical activity in children [25] and cut-points for sedentary intensity have been proposed for children [26]. The accelerometers were initialized to collect data in 60-sec epochs.

### Accelerometer data reduction

Participants aged 6 to 19 years with four or more valid days [24], one of which was a weekend day, were included in this analysis (Table 1). A valid day was defined as having 10 or more hours of wear time [24]. Wear time was determined by subtracting non-wear time from 24 hours. Non-wear time was defined as at least 60 consecutive minutes of zero counts, with allowance for two minutes of counts between zero and 100 [24]. For each minute, the level of movement intensity was based on cut-points corresponding to intensity level: sedentary = < 100 counts per minute (cpm) [26]; MVPA = ≥ 1,500 cpm [25]. Minutes of MVPA and sedentary time were summed for each day for each participant.

**Table 1:** Descriptive characteristics of the sample (mean ± standard deviation)

	**Boys**			**Girls**		
	**6 to 10 years**	**11 to 14 years**	**15 to 19 years**	**6 to 10 years**	**11 to 14 years**	**15 to 19 years**
Total sample (n)	369	256	184	340	248	211
Age (years)	8.2 ± 1.4	12.5 ± 1.0	17.0 ± 1.5	8.1 ± 1.3	12.3 ± 1.1	16.9 ± 0.1
Height (cm)	133.9 ± 10.4	158.9 ± 11.1	175.6 ± 7.6	131.6 ± 10.3	156.9 ± 7.8	166.2 ± 6.7
Weight (kg)	32.5 ± 9.4	52.1 ± 14.7	72.4 ± 18.1	29.9 ± 8.9	50.6 ± 11.6	62.5 ± 13.8
BMI (kg/m^2^)	17.8 ± 3.1	20.3 ± 3.9	23.4 ± 5.0	17.0 ± 3.1	20.4 ± 3.8	22.6 ± 4.4
Waist circumference (cm)	61.1 ± 9.8	70.6 ± 10.9	80.1 ± 12.9	57.9 ± 8.5	70.1 ± 10.0	75.4 ± 10.9
MVPA (average min·d^-1^)	69.4 ± 29.1	59.5 ± 29.4	53.1 ± 25.9	58.1 ± 22.6	47.2 ± 24.6	39.1 ± 23.0

*BMI *body mass index.

*MVPA *moderate-to-vigorous physical activity.

### Sedentary time variables

Sedentary time was calculated for all days, weekdays and weekend days. The total number of breaks in sedentary time was summed for each valid day and then averaged across the week, weekdays and weekend days. A break was considered as an interruption in sedentary time (lasting a minimum of one minute) in which there was a transition in accelerometer count from <100 cpm to ≥ 100 cpm.

To be defined as a prolonged sedentary bout, there had to be ≥80% of minutes below the 100 cpm cut-point (e.g., 16 out of 20 minutes or 32 out of 40 minutes) [1]. The bout stopped when <80% was below the 100 cpm cut-point or when there were ≥3 consecutive minutes ≥100 cpm or any observations ≥1500 cpm (cut-point for moderate intensity). Sedentary bouts lasting at least 20, 40, 60, 80, 100, 120 minutes were derived using this approach. Multiple lengths of sedentary bouts were derived to reflect a range of different sedentary behaviours such as watching a television show (30 minutes), watching a movie (1.5-2 hours), or playing video games (anywhere between 20 minutes and 2 hours). The choice of 80% as the criteria for sedentary minutes within a bout was purposeful to mimic real-world situations where largely sedentary pursuits (e.g., watching TV, doing homework) are often occasionally interrupted with light activity (e.g., to go to washroom, answer the phone, get a snack etc.).

### Body mass index and waist circumference

Height was measured to the nearest 0.1 cm using a ProScale M150 digital stadiometer (Accurate Technology Inc., Fletcher, USA) and weight was measured to the nearest 0.1 kg with a Mettler Toledo VLC with Panther Plus terminal scale (Mettler Toledo Canada, Mississauga, Canada). BMI was calculated as weight (kg) divided by height squared (m^2^). Children were categorized as not overweight/obese (which includes underweight and healthy weight) or overweight/obese according to age- and sex-specific cut-points [27]. Waist circumference was measured with a stretch-resistant anthropometric tape at the end of a normal expiration to the nearest 0.1 cm at the mid-point between the last rib and the top of the iliac crest [28].

### Blood pressure

Systolic and diastolic blood pressure were measured with the BpTRU™ BP-300 device (BpTRU Medical Devices Ltd., Coquitlam, British Columbia); an automated and validated [29,30] electronic monitor that uses an upper arm cuff. Six measurements were taken at 1-min intervals with the last 5 measurements used to calculate average blood pressure and heart rate [29]. The device automatically inflates and deflates the cuff and uses an oscillometric technique to calculate systolic and diastolic blood pressure.

### Non-HDL-Cholesterol

Non-HDL-cholesterol was calculated by subtracting HDL cholesterol, measured using a non-HDL precipitation method on the Vitros 5,1FS (Ortho Clinical Diagnostics), from total cholesterol [31]. Non-HDL cholesterol consists of very low density, low density, and intermediate density lipoprotein cholesterol and therefore reflects the cholesterol content of all apo B containing lipoproteins. Non-HDL cholesterol was chosen as the lipid marker because it is an important indicator of cardiovascular and diabetes risk among children and adolescents and is not reliant upon a fasted blood sample [32]. Blood samples were taken by a certified phlebotomist and were analyzed at the Health Canada Laboratory (Bureau of Nutritional Sciences, Nutrition Research Division). Other blood markers are available in the CHMS; however, the fasting requirement for some of these measures resulted in a marked reduction in the sample size when they are included. To ensure we had appropriate power for the primary purpose of this analysis, we included non-HDL-cholesterol as the sole blood marker.

### Statistical analysis

**Figure 1:**
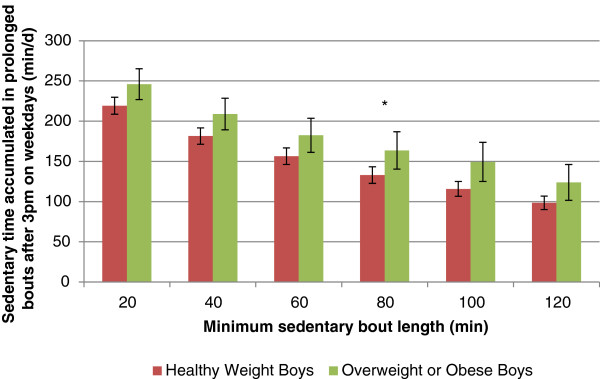
Sedentary time accumulated in prolonged bouts after 3 pm on weekdays in boys. *significant difference between healthy weight and overweight or obese.

**Figure 2:**
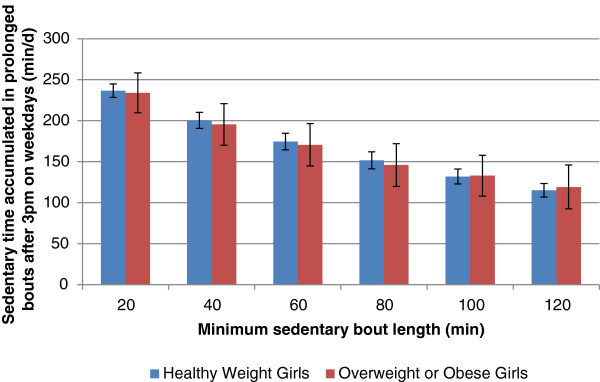
Sedentary time accumulated in prolonged bouts after 3 pm on weekdays in girls.

Differences between sex, age groups and BMI status were assessed using t-tests. Statistical significance was set at a *p* value of 0.05. It is important to note that the values presented from this analysis in Figures 1 and 2 represent the mean across the week for sedentary time accumulated in prolonged bouts. In other words, there are zeros included in the averaging (because not all individuals had bouts of each length on all days) which in the case of 120 minutes bouts, brings the mean time accumulated below 120 minutes.

Associations between sedentary time variables and BMI and waist circumference were assessed using regression analyses. BMI and waist circumference vary by sex and change with normal growth and maturation [27,33]. Age and sex were both significantly correlated with average daily minutes of MVPA and age was significantly correlated with average daily minutes of sedentary time. Therefore, all regression analyses were completed separately by sex and the following age categories: 6–10, 11–14 and 15 to 19 years. The choice of age categories was based on the sampling design of the CHMS. Linear regression models were run separately for each sex and age grouping and were adjusted for age, average daily minutes of MVPA on valid days, and accelerometer wear time. The wear time adjustment was specific to the time period being examined. For example, the models for weekdays after 3 pm where adjusted for wear time on weekdays after 3 pm. Eight separate regression models (sedentary time, breaks in sedentary time, prolonged bouts lasting at least 20, 40, 60, 80, 100, 120 minutes) were run for each time period: overall, weekdays after 3 pm and weekends. The *p*-value to reach statistical significance in the linear regression analyses was adjusted for the number of models run. In other words, to reach statistical significance, the regression *p* values had to be less than .006 (i.e., 0.05/8 = 0.006).

All statistical analyses were performed using SAS v9.1 (SAS Institute, Cary, NC) and were based on weighted data (to be representative of the Canadian population and to account for non-response bias) for respondents with at least four valid days. To account for survey design effects of the CHMS, standard errors, coefficients of variation, and 95% confidence intervals were estimated using the bootstrap technique [34-36]. Overweight and obese were collapsed into one category because we lacked statistical power to compare not overweight/obese (includes healthy weight and underweight), overweight and obese as 3 separate categories.

## Results

Descriptive characteristics of the sample are provided in Table 1. The analysis is based on 1,608 children and youth between the ages of 6 and 19 years. The sex split was even between boys (n = 809, 50.3%) and girls (n = 799).

### Sex and Age differences

Sex and age differences are presented in Table 2. On average, boys accumulated 508 minutes per day of sedentary time while girls accumulated 524 minutes per day. Boys accumulated more sedentary time on weekdays after 3 pm compared to girls, while boys had a lower number of breaks in sedentary time per day compared to girls. Sedentary time was higher in 11–14 year olds and 15–19 year olds compared to children aged 6–10 years. Girls aged 15–19 years accumulated more sedentary time overall and after school compared to boys of the same age.

**Table 2:** Descriptive sedentary time results (mean ± standard deviation), by age, sex and obesity status

	**Sedentary time (min/d)**	**Sedentary time on weekdays after 3 pm (min/d)**	**Sedentary time on weekends (min/d)**	**Breaks in sedentary time per day (number/d)**
**Boys**	**507.5 ± 90.8**	265.5 ± 65.8^*^	**490.9 ± 114.5**	81.2 ± 11.6^*^
*Age*				
6 to 10 years	445.5 ± 79.5	212.8 ± 52.0	440.5 ± 101.1	84.4 ± 10.3^*^
11 to 14 years	524.1 ± 76.9^**^	273.9 ± 58.0^**^	503.2 ± 103.3^**^	79.2 ± 11.2^*,**^
15 to 19 years	553.9 ± 77.0^*,**^	310.7 ± 44.7^*,**^	533.8 ± 126.4^**^	80.1 ± 13.6^**^
*BMI*				
Not overweight or obese	499.9 ± 88.4	259.4 ± 63.7	483.7 ± 115.0	81.2 ± 11.3
Overweight or obese	527.8 ± 94.6	281.7 ± 69.2^***^	509.7 ± 220.0	81.1 ± 12.6
**Girls**	**523.8 ± 91.6**	**277.1 ± 67.5**	**493.6 ± 106.7**	**85.4 ± 11.7**
*Age*				
6 to 10 years	446.1 ± 72.9	215.4 ± 52.1	428.7 ± 96.0	89.6 ± 10.4
11 to 14 years	526.8 ± 63.5^**^	275.0 ± 49.3^**^	503.2 ± 87.5^**^	84.7 ± 10.5^**^
15 to 19 years	582.1 ± 81.4^**^	326.3 ± 47.5^**^	538.4 ± 106.1^**^	82.6 ± 13.6^**^
*BMI*				
Not overweight or obese	523.5 ± 91.3	277.1 ± 68.3	495.4 ± 107.2	85.2 ± 11.5
Overweight or obese	524.6 ± 93.1	277.0 ± 64.2	487.1 ± 105.0	86.2 ± 12.5

^* ^significantly different to estimate for girls (p < .05).

^** ^significantly different to estimate for 6–10 year olds of same sex (p < .05).

^*** ^significantly different to estimate for not overweight or obese (p < .05).

BMI, body mass index.

### Body mass index differences

Differences by BMI status are represented graphically in Figure 1 for boys and in Figure 2 for girls. Overweight and obese boys accumulated more sedentary time after 3 pm on weekdays when compared to boys who are not overweight/obese (Table 2). Overweight and obese boys accumulated more sedentary time after 3 pm on weekdays as prolonged bouts lasting at least 80 minutes when compared to boys who are not overweight/obese (171 vs. 133 min∙d^-1^) (Figure 1). No sedentary time variables differed between girls who are overweight/obese and those who are not overweight or obese (Table 2; Figure 2).

### Regression analysis results

Prolonged bouts of sedentary time lasting at least 40 minutes, after 3pm on weekdays, were positively associated with waist circumference (ß = 2.23, *p* < .006) while prolonged bouts of sedentary time lasting at least 80 minutes was positively associated with both BMI (ß = 0.72, *p* < .006) and waist circumference (ß =1.76, *p* < .006) in boys aged 11–14 years. Each additional 60 minutes of sedentary time accumulated during the after school period was associated with a 1.4 kg∙m^-2^ higher BMI and a 3.4 cm higher waist circumference in 11–14 year old boys. Number of breaks in sedentary time, after 3pm on weekdays, was negatively associated with waist circumference (ß = –4.04, *p* < .006) in boys aged 11–14 years. No sedentary time variables were significantly associated with BMI or waist circumference in girls of any age or in boys aged 6–10 or 15–19 years. No sedentary time variables were associated with blood pressure or non-HDL cholesterol in boys or girls. The full results from the regression analyses are available as Additional file 1: Tables S1, Additional file 2: Tables S2, Additional file 3: Tables S3, Additional file 4: Tables S4, Additional file 5: Tables S5.

## Discussion

The objective of this study was to examine the association between accelerometer-measured sedentary time and health risk in children. Our analysis supports previous studies that found few or no significant associations between accelerometer-measured sedentary time and health risk in children [1,11-16]. This study is novel because it included a wider range of sedentary time variables than what has been previously considered that characterize the timing and patterning of how the sedentary time is accumulated. Further, the sedentary pattern variables were designed to be more reflective of real-world sedentary behaviour. For example, a limited number of short transitions into light activity were allowed to reflect real-life situations where individuals are sedentary for long periods but move around occasionally (i.e., to go to the washroom or answer the phone). Despite the inclusion of more comprehensive sedentary pattern variables, this study found few significant relationships with health risk and the associations observed were limited to boys aged 11–14 years.

In theory, excessive sedentary time is associated with negative health outcomes [4,37] and self-reported screen time is associated with elevated health risk in children [1-3]; however, the way we currently measure sedentary time with accelerometers does not consistently support this link. To date, the research linking accelerometer-measured sedentary time with health risk among children and youth has been mixed. It is therefore unclear whether a relationship exists only in some populations or if differences in analytical approaches explain the inconsistencies observed. There appears to be more evidence supporting a lack of relationship between accelerometer-measured sedentary time and health risk in children and youth [1,11-16] than there is supporting a relationship [8-10]. Adjustment for MVPA appears to attenuate significant associations between accelerometer-measured sedentary time and health risk [13,16], suggesting that MVPA is more powerful than total sedentary time at explaining the variance in health risk in children and youth. In our unadjusted regression models, sedentary time was associated with BMI and waist circumference in boys aged 6 to 14 and girls aged 6 to 10 years; however, after adjustment for MVPA, these associations remained significant only in 11–14 year old boys.

In 2008, Healy and colleagues published a paper that reported a significant association between number of daily breaks in accelerometer-measured sedentary time and lower metabolic risk in adults [17]. This work led researchers to question whether it is the pattern of how sedentary time is accumulated, rather than simply the total volume of sedentary time, which matters for health. Do frequent interruptions in sedentary time attenuate the health risk that sedentary time imposes? Does this relationship apply in both children and adults? Carson and Janssen found no significant associations between breaks in sedentary time or prolonged bouts of sedentary time lasting 30 minutes with cardio-metabolic risk factors in a large sample of American children [1]. Number of breaks in sedentary time was only associated with waist circumference in 11–14 year old boys in the present analysis. We included an additional layer of complexity by examining sedentary time, breaks and prolonged bouts of sedentary time during periods of discretionary free time: weekends and after school. We hypothesized that sedentary time accumulated during these periods would better discriminate between children engaging in healthy and unhealthy levels of sedentary behaviour when compared to simply examining overall sedentary time. We observed no significant associations between the patterns of sedentary time accumulated on weekends and health risk in children; however, some relationships emerged when we examined sedentary time accumulated during the after school period. Interestingly, we only observed significant associations in boys aged 11 to 14 years of age when the regression models were adjusted for age, MVPA and accelerometer wear time.

It is difficult to speculate why we observed significant findings in boys and not girls. It is possible that more overweight and obese boys in this sample were engaging in prolonged bouts of sedentary time after school, a finding consistent with previous research that has found that boys spend considerably more time in specific sedentary behaviours such as video game playing [38-40]. Average daily sedentary time and weekend sedentary time did not differ between boys and girls while sedentary time accumulated after 3 pm on weekdays was higher in boys compared to girls (277 vs. 266 minutes). Significant differences between boys who are not overweight/obese and overweight/obese boys were observed in the sedentary variables; however, no such differences were observed in girls. For example, there was virtually no difference in average daily sedentary time between girls who are not overweight/obese versus those who are (524 vs. 525 min∙d^-1^) while a more marked difference existed between boys who are not overweight/obese versus those who are (500 vs. 528 min∙d^-1^). In Figure 1, a distinction between boys who are not overweight/obese and those who are can be observed across all bout lengths; however the difference is only statistically significant when the bout length is at least 80 minutes long. By comparison, no difference is noticeable by overweight/obesity status in girls and there is more cross-over in the error bars in girls (Figure 2). Similarly, no significant associations emerged in girls in the regression analyses while in 11–14 year old boys, prolonged bouts of sedentary time lasting at least 80 minutes, accumulated during the after school period were associated with both BMI and waist circumference. Explaining why significant associations were observed in 11–14 year olds boys but not those who were 6–10 or 15–19 years is not easy. In a large sample of US children, Sisson and colleagues observed an increase in screen time with age from 2 to 15 years [41]. In the Health Behaviour and School Aged Children Survey, the Canadian data show that screen time increases from age 11 to 15 years [42] with the peak occurring in grade 9 (approximately 14 years) [43]. These large data sets suggest that the 11–14 year old age group may be an age range where screen time habits change significantly, thus increasing the amount of variation (and likelihood to find significant associations) in this variable.

The lack of evidence linking accelerometer-measured sedentary time with health risk in children is counter-intuitive given the consistent observation that screen time, a key contributor to total sedentary time, is associated with health risk [1-4]. One of the fundamental differences between self-reported screen time and objectively measured sedentary time is that the former is capturing one specific activity while the latter is capturing screen time in addition to many other sedentary behaviours. Much of the time accumulated as “sedentary” represents normal aspects of day-to-day life therefore capturing every minute in a day that is sedentary, as accelerometers do, may dilute the associations between specific sedentary behaviours (e.g., watching television) and health risk. It is possible that some forms of sedentary behavior (e.g., screen time, long car or bus travel) are associated with negative health outcomes while other forms of sedentary behavior (e.g., eating, reading, resting, socializing etc.) are not. Similarly, data reduction procedures used in accelerometry analysis (e.g., 10 hour wear time criteria) were developed to accurately capture MVPA and whether they are appropriate for sedentary behaviour research questions is unknown. For example, it has been suggested that wear time has a disproportionate impact upon estimates of sedentary time compared with MVPA [44]. Teasing out which sedentary behaviours beyond screen time are associated with negative health outcomes represents an important area for future research.

The sedentary behaviours that are of known public health concern in children and youth (e.g., excessive levels of screen time) typically last for extended periods of time (i.e., up to several hours at once). This reality was the motivation behind the way prolonged bouts of sedentary time were defined in the present analysis. Had we used a strict definition of what ended about (i.e., any transition out of sedentary) then our longest bout length would have been very short (e.g., 10 minutes) and thus not representative of one of the key sedentary behaviours that we were interested in capturing. The allowance of a modest amount of light intensity movement within the prolonged sedentary bouts was therefore purposeful and allowed much longer bout lengths to be examined (up to 2 hours). Number of breaks per day, also assessed in this analysis, is an important aspect of sedentary behaviour patterns. Given that we and others [1] have not consistently observed significant associations between number of breaks per day and health risk, it is important to look at alternative pattern variables such as prolonged bouts. Further, the extension of bout length in the present analysis was important to build off the only other published work that examined prolonged bouts up to 30 minutes in children and youth [1].

It is possible that the true health effect of sedentary time is attenuated by limitations with the data and analysis. Possible limitations that dilute the possibility of observing a true relationship include: i) the cross-sectional nature of the data, ii) non-response bias, iii) the possibility that the findings in 11–14 year olds boys reflect Type 1 error. As described in the methodology, the non-response bias is adjusted for in the data. We attempted to minimize the likelihood of Type 1 errors in the regression analyses by adjusting the p-value for significance from .05 to .006. Other limitations include the lack of ability to confirm precisely when children were finished school. We examined the period after 3 pm on weekdays [45] based on the assumption that most kids would finish school sometime between 2-4 pm. Accelerometers are limited in their ability to capture postural changes (i.e., cannot differentiate between sitting and standing) and are therefore limited in their ability to measure sedentary before as well as other tools which encompass an inclinometer in addition to an accelerometer. No significant associations were observed between sedentary time variables and blood pressure or non-HDL cholesterol and this may be due to it likely being more difficult to detect meaningful differences in biomarkers in children and youth than adults because younger people are more distal to pathophysiological developments. A similar examination on a population of high-risk children (e.g., overweight or obese or with a family history of cardio-metabolic disease) may lead to different findings as these children would be more likely to exhibit abnormalities in blood markers and blood pressure. Finally, examination of interaction and confounding effects was limited because the number of variables (including interaction terms) that can be tested within the CHMS data set is limited by the available degrees of freedom.

## Conclusions

Sedentary time accumulated during the after school period was associated with BMI and waist circumference, independent of MVPA, in boys aged 11 to 14 years. No sedentary behaviour variables were independently associated with any health markers in older or younger boys or in girls of any age. Future studies should consider examining more comprehensive sedentary time pattern variables when attempting to elucidate the relationships between sedentary time and health risk in children and youth.

## Competing interests

The authors declare that they have no competing interests.

## Authors’ contributions

RCC conceived the manuscript, formed the research team, directed the analysis, led the writing. DG was involved in the conception the manuscript, completed the analysis and contributed to the writing. IJ contributed to the writing and provided critical review of the analysis. SLW was involved in the conception of the manuscript, contributed to the writing and provided critical review of the analysis and writing. TJS, VC and MST contributed to the writing and provided critical review of the writing. All authors read and approved the final manuscript.

## Pre-publication history

The pre-publication history for this paper can be accessed here:

http://www.biomedcentral.com/1471-2458/13/200/prepub

## Supplementary Material

Additional file 1: Table S1 Associations between sedentary time variables and body mass index, presented by sex and age groups. Click here for file

Additional file 2: Table S2 Associations between sedentary time variables and waist circumference, presented by sex and age groups. Click here for file

Additional file 3: Table S3 Associations between sedentary time variables and systolic blood pressure, presented by sex and age groups. Click here for file

Additional file 4: Table S4 Associations between sedentary time variables and diastolic blood pressure, presented by sex and age groups. Click here for file

Additional file 5: Table S5 Associations between sedentary time variables and non-HDL cholesterol, presented by sex and age groups. Click here for file
